# Effects on Nitric Oxide Production of Urolithins, Gut-Derived Ellagitannin Metabolites, in Human Aortic Endothelial Cells

**DOI:** 10.3390/molecules21081009

**Published:** 2016-08-02

**Authors:** Valentina Spigoni, Pedro Mena, Monia Cito, Federica Fantuzzi, Riccardo C. Bonadonna, Furio Brighenti, Alessandra Dei Cas, Daniele Del Rio

**Affiliations:** 1 Endocrinology and Metabolism, Department of Clinical and Experimental Medicine, University of Parma, Parma 43126, Italy; valentina.spigoni@unipr.it (V.S.); monia.cito@hotmail.it (M.C.); federica.fantuzzi1@gmail.com (F.F.); riccardo.bonadonna@unipr.it (R.C.B.); alessandra.deicas@unipr.it (A.D.C.); 2 The Laboratory of Phytochemicals in Physiology, Department of Food Science, University of Parma, Parma 43125, Italy; pedromiguel.menaparreno@unipr.it (P.M.); furio.brighenti@unipr.it (F.B.); 3 Division of Endocrinology, Azienda Ospedaliero-Universitaria of Parma, Parma 43126, Italy

**Keywords:** urolithin, ellagic acid, microbiota metabolites, endothelial cells, endothelial nitric oxide synthase, endothelial function, atherosclerosis, peripheral metabolism

## Abstract

The consumption of foodstuffs yielding circulating compounds able to maintain endothelial function by improving nitric oxide (NO) bioavailability can be considered as an effective strategy for cardiovascular disease prevention. This work assessed the in vitro effects of urolithin A, urolithin B, and urolithin B-glucuronide, ellagitannin-derived metabolites of colonic origin, on NO release and endothelial NO synthase (eNOS) activation in primary human aortic endothelial cells (HAECs). Urolithins were tested both individually at 15 μM and as a mixture of 5 μM each, at different time points. The biotransformation of these molecules in cell media due to cell metabolism was also evaluated by UHPLC-MS^n^. The mix of urolithins at 5 μM significantly increased nitrite/nitrate levels following 24 h of incubation, while single urolithins at 15 μM did not modify NO bioavailability. Both the mix of urolithins at 5 μM and urolithin B-glucuronide at 15 μM activated eNOS expression. All urolithins underwent metabolic reactions, but these were limited to conjugation with sulfate moieties. This study represents a step forward in the understanding of cardiovascular health benefits of ellagitannin-rich foodstuffs and backs the idea that peripheral cells may contribute to urolithin metabolism.

## 1. Introduction

The endothelium is a key regulator of vascular homeostasis, representing not only a selective barrier, but also modulating circulating blood cell adhesion, smooth muscle cell proliferation, and inflammation. In addition, it plays a crucial role in the maintenance of vascular tone by regulating the fine-tuned balance between vasodilation and vasoconstriction [1]. Nitric oxide (NO) is the most important endothelium-dependent vasodilator produced by the endothelial nitric oxide synthase (eNOS) in blood vessels. Decreased eNOS activation and the subsequent reduction in NO bioavailability are the main determinants of endothelial dysfunction, an early step in the development of atherosclerosis [2]. Endothelial dysfunction is a reversible process and effective strategies in maintaining or improving vascular homeostasis are of critical interest for cardiovascular disease (CVD) prevention.

Phenolic compounds may exert protective actions against atherosclerosis and other CVDs [3,4,5,6]. Recently, a randomized, controlled, double-masked trial showed a significant reduced risk of coronary heart disease (CHD), myocardial infarction, and death from CHD and CVD after bi-daily ingestion of cocoa flavanols [7]. This same study reported a significant improvement in endothelial function (measured as flow-mediated vasodilation (FMD)). Consumption of polyphenol-rich cranberry juice also improved endothelial function in patients with coronary artery disease [8] and healthy men [9]. While it may seem obvious to ascribe the effects of foodstuffs rich in (poly)phenolic compounds on endothelial function to their phenolic fraction, the relationship between the metabolites in circulation and the observed effects is not usually tackled [8]. Interestingly, current literature accounts for a positive change in addressing this issue [9]. On the other hand, cell-based evidence on the modulation of endothelial function by phenolic compounds is far from explaining the effects observed in humans. Different cell studies carried out with phenolic compounds have shown induced NO release through a promotion of eNOS expression in human endothelial cells [10,11]. However, metabolic transformations or the dose of circulating phenolic metabolites have not been really taken into account in the past. In most of the cases, in vitro studies have applied molecules never appearing in vivo, or concentrations far from those achievable in the context of a normal diet. Currently, the number of cell studies performed with phenolic circulating metabolites at physiological concentrations is growing and outcomes point to a modulated vascular reactivity and improved NO bioavailability [12,13].

Ellagitannins are hydrolyzable tannins present in pomegranate, walnuts, berries such as raspberries, strawberries, and blackberries, and oak-aged wines [14]. Once consumed, ellagitannins are hydrolyzed into ellagic acid, which is further metabolized by the intestinal microbiota to produce urolithins [15]. Urolithins are hydroxylated 6*H*-dibenzo[*b*,*d*]pyran-6-one structures that may undergo colonic/hepatic transformation upon absorption and appear in circulation as sulfate, glucuronide, and methyl metabolites at concentrations in the low micromolar–high nanomolar range [14,15,16]. Although the evidence is limited, some experimental studies have demonstrated the anti-inflammatory and anti-proliferative effects of urolithins, as well as the ability of some food matrixes rich in ellagitannins to improve endothelial function [17,18,19,20,21,22,23,24,25,26,27]. Nevertheless, the potential role of urolithins in modulating NO-related endothelial cell function has been scarcely investigated to date [28].

The aim of this work was to assess the in vitro effects of urolithin A (Uro A, dihydroxy-urolithin), urolithin B (Uro B, monohydroxy-urolithin), and urolithin B-glucuronide (Uro Bgluc) on endothelial function, in terms of eNOS activation and NO release, in primary human aortic endothelial cells (HAECs). These molecules were tested both individually, at 15 μM, and as a mixture of 5 μM each. The biotransformations of these ellagitannin-derived metabolites in the tested cell model, as a consequence of cell metabolism, were also evaluated.

## 2. Results

### 2.1. NO Production in Human Aortic Endothelial Cells

To establish whether urolithins could modulate vascular reactivity in a non-inflammatory scenario, the production of NO was studied in non-stimulated conditions. Urolithins were added individually at 15 μM, in line with previous works [18], and pooled at 5 μM each (5 μM of Uro A, 5 μM of Uro B, and 5 μM of Uro Bgluc, named “mix”), similarly to Mele et al. [25], to obtain the same final metabolite concentration.

Endothelial NOS activation by “classical” cues (such as insulin and bradykinin) is known to occur acutely (after a few minutes) in endothelial cells [29,30]. In addition, since NO is a gaseous molecule with a short half-life, nitrite (NO^2−^) and nitrate (NO^3−^) levels—which represent the stable degradation products of NO accumulating in supernatants of cell culture—were assessed at 5 min and at 24 h as an integrated measure of NO production [31,32,33].

**Figure 1:**
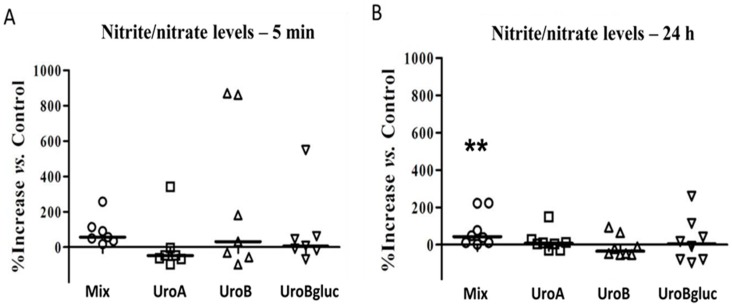
Variation in nitrite/nitrate levels in the supernatant of HAECs cultured at 5 min (**A**) and 24 h (**B**) with respect to control condition (0.1% DMSO). Mix: urolithin mix at 5 μM each; uro A: urolithin A; uro B: urolithin B; Uro Bgluc: urolithin B-glucuronide. *n* = 7; ** significant at *p* < 0.01 vs. control. Each experimental condition is represented by a specific symbol (Mix: circles, Uro A: squares, Uro B: triangles, Uro Bgluc: inverted triangles) and bars represent the median values.

None of the single urolithins at 15 μM modified NO metabolite production in HAECs following both 5 min and 24 h of incubation, compared to vehicle culture conditions (Figure 1). However, the mix of urolithins significantly increased nitrite + nitrate levels in HAECs following 24 h of incubation (*p* < 0.01). This may be the result of the higher efficacy of urolithins pooled at 5 μM, than when tested individually at 15 μM. On the other hand, it is worth mentioning the high variability observed among independent experiments, which could be attributed to the nature of these primary cells.

### 2.2. eNOS Activation in Human Aortic Endothelial Cells

**Figure 2:**
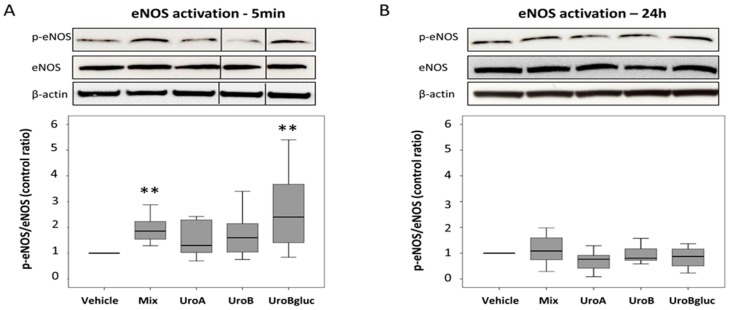
eNOS activation in HAECs cultured at 5 min (**A**) and 24 h (**B**) with respect to control condition (0.1% DMSO). eNOS activation was assessed by Western blotting and expressed as the ratio of phosphorylated-eNOS (p-eNOS) to total eNOS protein expression. Beta-actin has been used as loading control. One representative experiment is reported, bars represent median values (*n* = 7). Mix, urolithin mix at 5 μM each; uro A, urolithin A; uro B, urolithin B; and Uro Bgluc, urolithin B-glucuronide. ** significant at *p* < 0.01 vs. control.

In order to clarify the role of eNOS in the observed NO secretion, eNOS activation at the same time points (5 min and 24 h) was assessed. Exposure to mixed urolithins increased eNOS activation at 5 min (1.8-fold increase vs. control) but not at 24 h (Figure 2), justifying the increase in NO levels at the later time point (24 h). Uro A and Uro B at 15 μM did not exert any statistically significant effect on eNOS expression either at 5 min or at 24 h. In contrast, a significant 2.4-fold increase in eNOS activation compared to the control was observed when HAECs were incubated in the presence of Uro Bgluc 15 μM for 5 min.

### 2.3. In Vitro Metabolism of Urolithins in HAECs

Cellular metabolism is a key point to be considered when dealing with the biological activity of phenolic compounds, since the ability of some cell types to uptake and metabolize phenolic compounds or their metabolites may influence the biological effects of the tested parent compounds. Gastrointestinal tract cells are recognized to be pivotal in the absorption, metabolism, and bioactivity of phenolics [4,6], but other cell types may also exert metabolic structural modifications. This peripheral metabolism of phenolic compounds, and in particular of their circulating metabolites, has been scarcely studied [34]. Recently, some progress has been achieved in the case of urolithins by using cardiomyocytes and cardiac fibroblasts [26], and macrophages and human umbilical vein endothelial cells (HUVECs) [25]. However, the intracellular metabolism of urolithins by human aortic endothelial cells was still not reported.

**Figure 3:**
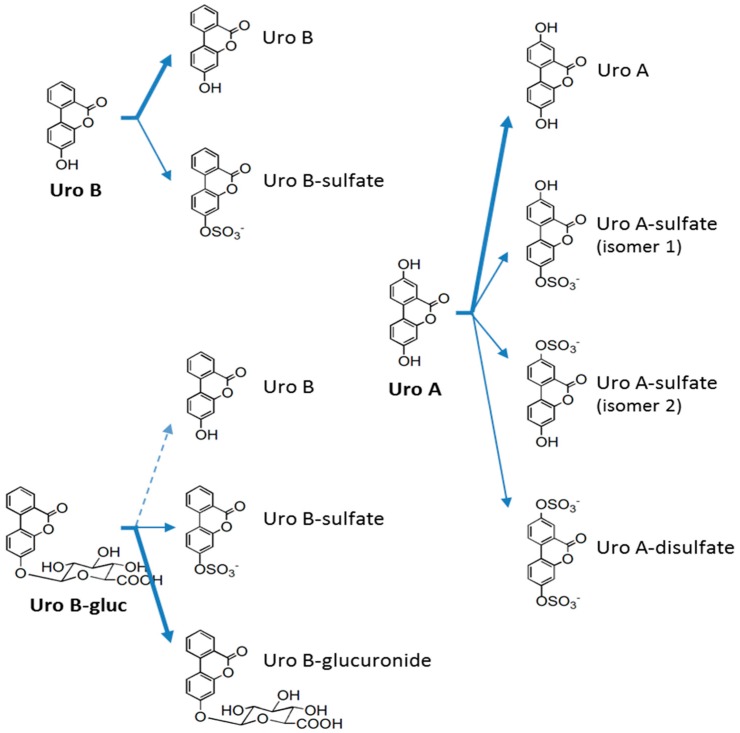
Metabolic biotransformations occurring in human aortic endothelial cells and compounds present contemporaneously in the cell media at 24 h. Bold arrows indicate major pathways, which correspond to incubated urolithins; normal arrows, newly-formed metabolites; and dotted arrows, spontaneous deglucuronidation due to cell media conditions.

In order to verify the peripheral metabolism of urolithins in HAECs and to get insights into the real molecules behind the effects observed on NO bioavailability, cell media of the performed experiments were analyzed at the beginning and end of each incubation time. Culture media and non-incubated cell media were used to test urolithin stability and as the control, respectively. Different transformations that might take place (conjugation with sulfate, glucuronide, methyl, glutathione, and cysteine moieties, quinone formation, and (de)hydroxylation) were monitored by a specific and sensitive ultra-high performance liquid chromatography coupled to mass spectrometry (UHPLC-MS^n^) analysis [25]. Four newly formed metabolites were identified according to their fragmentation patterns and retention times (Table 1). All urolithins underwent metabolic reactions, but were basically limited to conjugation with sulfate moieties (Figure 3). Unfortunately, newly formed metabolites were not quantified as reference compounds were not commercially available. Thus, peak area values were considered when comparisons were made.

**Figure 4:**
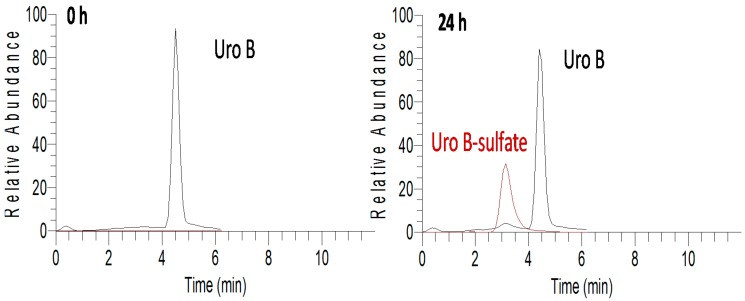
Chromatograms of compounds present in cell media at 0 and after 24 h of incubation with Uro B, extracted in selected ion-monitoring mode. Areas of the metabolites should not be considered as quantitative indicators since ionization efficiencies of the individual compounds may be different.

Both Uro A and Uro B were stable under cell culture conditions, while Uro Bgluc underwent a weak deglucuronidation (~1%). Despite all the tested molecules undergoing metabolic reactions, the concentration of the incubated molecules, both individually and mixed, remained almost unaltered after 24 h (*p* > 0.05), indicating a quite negligible conversion rate. The formation of sulfated metabolites in the cell culture was detected at 24 h but not at 5 min after urolithin incubation. In the case of Uro B-sulfate, a higher rate of sulfation was observed for Uro B than for Uro Bgluc (about nine-fold higher, *p* < 0.001) (Figure 4). With regard to metabolites derived from Uro A, mono-sulfated isomers were almost equally concentrated, while Uro A-disulfate was detected only at trace levels. Finally, it should be noted that the co-presence in the mix of the three urolithins (Uro B, Uro Bgluc, and Uro A) did not alter the metabolic activity of HAECs, since the metabolites identified when the cells were incubated with single compounds were also detected after exposure to the mix.

## 3. Discussion

Impairment in eNOS activation preludes endothelial dysfunction and atherosclerosis. Therefore, the demonstration of a benefit of ellagitannin-derived metabolites on endothelial function could be considered a mechanism through which dietary ellagitannins may contribute to protection against atherosclerotic processes. An in vivo study revealed that pomegranate fruit extract rich in ellagitannins significantly decreased the expression of vascular inflammation markers (thrombospondin and cytokines) and increased plasma NO levels in obese Zucker rats [17]; while in another study, the administration of 100–300 mg/kg/day for four weeks of pomegranate juice extract to diabetic rats treated with angiotensin II decreased arterial blood pressure and improved biomarkers linked to oxidative stress [35]. While some in vitro studies have shown anti-atherosclerotic effects of ellagitannins in endothelial cells by exposing culture media to full plant extracts or to single parent plant compounds at rather high concentrations [15], the present study was the first in assessing the effects of a mix of circulating ellagitannin metabolites at physiologically attainable concentrations on endothelial cell function. Results showed how a mixture of 5 μM of urolithins A, B, and B-glucuronide, the most representative ellagitannin-derived metabolites, increased NOx bioavailability by eNOS activation in human aortic endothelial cells. The fact that the mixture of urolithins seemed to be more bioactive in improving NO release compared to its individual constituent compounds is of critical interest. It may suggest that the reported benefits of an ellagitannin-rich diet could be due to the activities of metabolites working in combination, as it would potentially occur following dietary exposure, and not merely as the exact consequence of a single metabolite. This assumption has been recently proposed in an in vitro study in which combinations of flavonoid-derived metabolites significantly reduced TNF-α to a greater extent than their precursors or single metabolites alone [36]. Although the effects of polyphenol metabolites on NO release might be magnified in insulin-stimulated conditions [37], we decided to carry out the experiments in a basal unstimulated experimental setting, in order to avoid potential confounding factors. We cannot exclude that the presence of insulin might have amplified the observed results. Furthermore, it must be considered that nitrite/nitrate assessment, although commonly used, is an indirect measure of NO levels, and therefore per se constitutes a limitation.

Uro Bgluc at 15 μM increased eNOS protein activation in the tested cell model. However, increased eNOS activation following Uro Bgluc incubation was not accompanied by an increase in NO levels. This effect is not easily justified, and one can speculate that the urolithin mix could lead to more persistent enzyme activation than Uro Bgluc, with a subsequent major increase in NO production. Hypothetically, this persistent activation of eNOS would have been detectable in the time lapse between the 5 min and the 24 h time points, which was not investigated in this study. The 15 μM concentration of metabolites used in this study is comparable to that obtained in a study after the ingestion of 1 L of pomegranate juice containing 4.37 g of punicalagins for five days, in which plasma urolithin levels reached a maximum concentration of 18.6 μM [38]. Although lower concentrations of circulating Uro Bgluc are expected after normal consumption of ellagitannin-rich foodstuffs, these results are of clinical interest since they may generate interesting prospects in terms of cardiovascular health promotion and future research. Nevertheless, it should be kept in mind that current evidence accounts for a large inter- and intra-individual variability in the levels of circulating urolithins, indicative of differences in the colonic microbiota responsible for ellagitannin degradation [39]. This fact may certainly hamper the settings of mean values for ellagitannin-derived circulating metabolites, as well as dietary guidelines for ellagitannin consumption.

Importantly, our study was performed in human cultured endothelial cells (HAECs), which represent a primary cell population directly exposed to circulating urolithin metabolites and involved in the pathophysiology of atherosclerosis. In the same cellular model and in accordance with our data, a very recent work showed that Uro A attenuated ox-LDL–induced endothelial dysfunction by an up-regulation of NO expression and eNOS mRNA expression [28]. In addition, Uro A and its glucuronide Uro A-glucuronide showed anti-inflammatory effects by reducing monocyte adhesion, endothelial cell migration and expression of pro-inflammatory chemokines [18].

This mounting evidence may serve to establish the role of ellagitannin metabolites in the promotion of endothelial health. By showing positive effects of these metabolites in improving vascular endothelial cell function in vitro, these results also expand previous knowledge of the anti-inflammatory and chemopreventive effects of urolithins [18,19,20,21,22,24,25,26,27]. Actually, most of the studies conducted so far clearly demonstrated the anti-inflammatory effects of urolithins in THP-1 [25] and RAW macrophages [40]. Importantly, in an in vivo study Uro A supplementation was able to abrogate the NO production by avoiding iNOS induction in the colonic mucosa of a colitis rat model [24]. The putative molecular pathway underlying the capacity of urolithins to increase NO_x_ production is beyond the scope of our study. However, on a purely speculative base, urolithins contain hydroxyl groups, which may potentially generate ROS through auto-oxidation. This, in turn, may induce the activation of Src kinases (i.e., Akt, AMPK, and eNOS) leading to NOx production, as previously demonstrated for other polyphenols, i.e., hesperidin [41], epigallocatechin gallate [31], and red wine polyphenols [42], as a putative mechanism for improved endothelial function in vitro.

In order to fully elucidate the real metabolites behind the effects showed in the HAECs, the possible biotransformations of urolithins in cell media were assessed. Actually, urolithins have been shown to undergo extensive metabolism in peripheral cells and not only at colonic and hepatic levels [25,26]. Our research group has described how both Uro A and Uro B are sulfated by both HUVECs and THP-1–derived macrophages [25]. The modifications carried out by cardiomyocytes and cardiac fibroblasts include, besides sulfation, glucuronidation and, in the case of Uro Bgluc, deglucuronidation [26]. The present results indicate sulfation as the prevalent modification taking place in urolithins incubated with HAECs. Regarding all these outcomes, cell-mediated sulfation seems to be a common metabolic step undergone by urolithins in peripheral cells of the cardiovascular system. Nonetheless, with glucuronidation considered to be the main reaction occurring in vivo after ellagitannin consumption [38,43], deglucuronidation must happen before sulfation, at least of Uro Bgluc. Obviously, the quantitative levels or proportions of these newly formed metabolites, even in relation to their precursors, should be taken with caution, as without analytical standards one cannot guarantee ionization efficiencies of the structures will be similar.

Metabolic transformations in peripheral cells may be relevant for the understanding of the compounds and mechanisms responsible for the bioactivity of phenolic compounds. In the particular case of urolithins, different phase II conjugates (with glucuronide, sulfate, and methyl moieties) of the same chemical scaffold may cause different biological responses [44,45]. Deglucuronidation of quercetin-glucuronide has been reported to be a crucial, positive step in the anti-inflammatory properties of quercetin on lipopolysaccharide-stimulated macrophages [46,47]. On the contrary, quercetin deglucuronidation reduces eNOS protein and gene expression in HUVECs and, thus, might cause a potentially deleterious impairment in the endothelial function response [48]. However, these metabolic reactions should not be considered as generalized for all phenolic metabolites. For instance, no deglucuronidation was observed when differently activated macrophages were exposed to naringenin-glucuronide isomers (naringenin-7-*O*-glucuronide and narigenin-4′-*O*-glucuronide) at concentrations coherent with dietary exposure [49]. Similarly, major flavan-3-ol metabolites ((−)-epicatechin-3′-glucuronide, (−)-epicatechin-3′-sulfate, 3′-*O*-methyl-(−)-epicatechin-5-sulfate, and 3′-*O*-methyl-(−)-epicatechin-7-sulfate) were not metabolized when incubated with HUVECs, while exposure of free epicatechin to HUVECs entailed its intracellular metabolism to 3′-*O*-methyl-(−)-epicatechin-7-sulfate and 3′-*O*-methyl-(−)-epicatechin-7-glucuronide [34].

## 4. Materials and Methods

### 4.1. Chemicals

Uro A, Uro B, and Uro Bgluc were provided by Olivier Dangles (INRA, Avignon, France), and synthesised according to what described in Tognolini et al. [50].

### 4.2. Cell Culture

HAECs (purchased from GIBCO Life Technologies, Carlsbad, CA, USA) were cultured in complete EGM-2 Bullet Kit (Lonza, Basel, Switzerland) at 37 °C in a 5% CO_2_ humidified incubator and used at passages 3–7 in all the experiments. HAEC were seeded in T25 flasks at a density of 1 × 10^4^ cells/cm^2^. On reaching 70%–80% confluence, cells were cultured overnight in serum free-media and then incubated with urolithins or with vehicle (0.1% DMSO as negative control) for 5 min and 24 h. Uro A, Uro B and Uro Bgluc were incubated at a concentration of 15 μM. A mixture of urolithins at 5 μM each was also added to the culture medium in order to test the combined effects of metabolites.

### 4.3. Nitrite Analysis

Total nitrite (nitrate and nitrite) produced by cells was measured in the supernatants by using a nitrate/nitrite assay kit (Cayman Chemical Company, Ann Arbor, MI, USA), following manufacturer’s instructions. This assay determines NO concentrations based on the enzymatic conversion of nitrate to nitrite by nitrate reductase. The reaction is followed by colorimetric detection of nitrite. A standard curve was performed to quantitate nitrite concentration in each assay. Seven independent experiments were performed in triplicate for each condition and results were expressed as nitrite percentage increase in urolithin-treated cells compared to vehicle.

### 4.4. Total Protein Extraction

Following treatments, HAEC were washed twice with PBS, detached from the flasks by using trypsin-EDTA, centrifuged and stored at −20 °C. Mammalian Protein Extraction Reagent (M-PER, Thermo Fisher Scientific, Milano, Italy) containing Halt™ Protease and Phosphatase Inhibitor (Single-Use Cocktail, Thermo Scientific) was added to the cell pellets and proteins were extracted by gentle shacking for 10 min followed by centrifugation at 13,000× *g* for 15 min in order to remove cell debris. Protein quantification was determined according to the Bio-Rad Protein Assay (Bio-Rad Laboratories, Hercules, CA, USA) using bovine serum albumin (BSA) as standard.

### 4.5. Western Blotting

A total of 40 μg of proteins were separated by 4%–12% pre-cast polyacrylamide gel (NuPAGE^®^ Novex^®^ 4%–12% Bis-Tris Gels, Thermo Scientific) and transferred to nitrocellulose filters. Membranes were blocked with 5% skim milk (Membrane Blocking Agent, GE Healthcare, Chalfont St Giles, UK) in Tris-buffered saline solution containing 0.1% Tween-20 and incubated overnight at 4 °C with rabbit anti-human phospho-eNOS^Ser1177^ (1:1000), eNOS (1:1000) or β-actin (1:10,000) (Cell Signaling Technology, Boston, MA, USA) antibodies. Membranes were then incubated with Horseradish peroxidase-labeled goat anti-rabbit secondary antibody (Cell Signaling) and detected by chemiluminescence using ECL western Blotting Detection Reagent (GE Healthcare, Chalfont St Giles, UK). Images were scanned and quantified using ImageJ software (Version 1.48, National Institutes of Health, MD, USA). Activation of eNOS was expressed as a ratio of p-eNOS to eNOS (which detects both phosphorylated and non-phosphorylated forms).

### 4.6. UHPLC-MS^n^ Analysis of Cell Media

Cell media supernatants were collected and analyzed by UHPLC-MS^n^ to determine the stability and intracellular metabolism of the urolithins in cell culture. Cell media was extracted according to Sala et al. [26] and analyzed according to Mele et al. [25]. Briefly, samples were analysed using an Accela UHPLC 1250 equipped with a linear ion trap-mass spectrometer (LTQ XL, Thermo Fisher Scientific Inc., San Jose, CA, USA) fitted with a heated-electrospray ionization probe (H-ESI-II; Thermo Fisher Scientific Inc.). Separations were performed using a XSELECTED HSS T3 (50 × 2.1 mm), 2.5 μM particle size (Waters, Milford, MA, USA), with an injection volume of 5 μL, column oven temperature of 30 °C and elution flow rate of 0.2 mL/min. The initial gradient was 75% of 0.1% aqueous formic acid and 25% acetonitrile (in 0.1% formic acid), reaching 80% acetonitrile at 6 min. The MS conditions included: capillary temperature of 275 °C and source heater temperature of 250 °C, sheath gas flow of 40 units, auxiliary and sweep gas of five units, source voltage of 3 kV and capillary voltage and tube lens of −5 and −68 V, respectively. Analyses were carried out using full scan, data-dependent MS^3^ scanning from *m*/*z* 100 to 600, with collision induced dissociation (CID) equal to 35 (arbitrary units). Pure helium gas was used for CID. Data processing was performed using Xcalibur software (Version 2.2) from Thermo Scientific.

### 4.7. Statistical Analysis

Due to significant deviations from normality, the exact version of Friedman test was used to investigate differences among culture conditions. Differences between pairs of groups were identified using paired Wilcoxon test with Bonferroni correction for multiplicity (*p* < 0.0125).

Statistical significance was declared at *p* < 0.05 (two-sided). SPSS 21 (IBM, Armonk, NY, USA) and Prism 4 (Graphpad, La Jolla, CA, USA) were used for statistical analyses and graphs.

## 5. Conclusions

This study represents a step forward in the understanding of the cardiovascular health benefits of ellagitannin-rich foodstuffs. It evidenced how urolithin B glucuronide at 15 μM and a mixture of urolithins A, B, and B-glucuronide at 5 μM each can be effective in improving aortic endothelial cell function in vitro. These results also support the idea that peripheral cells may contribute significantly to urolithin metabolism.

Although promising, these in vitro results on the biological activity of urolithins should be cautiously translated to in vivo physiological conditions. Future randomized, controlled human interventions, properly designed to take into account the late production of these ellagitannin-derived metabolites, are required to fully define the cardioprotective features of ellagitannin-rich foodstuffs. Similarly, novel dynamic models of cell culture adhering to transient concentrations of phenolic metabolites should be investigated.

## Figures and Tables

**Table 1:** Urolithins detected in endothelial cells.

Compound	Derived Metabolite	Retention Time (min)	[M − H]^−^ (*m*/*z*)	MS^2^ Ion Fragments (*m*/*z*)	MS^3^ Ion Fragments (*m*/*z*)
*Urolithin B*	Uro B	4.50	211	167, 182	
	Uro B-sulfate	3.71	291	*211* ^1^	167
	Uro B-glucuronide	2.45	367	*211*, 175	167
*Urolithin A*	Uro A	3.19	227	183, 159, 199	
	Uro A-sulfate isomer 1	1.58	307	*227*	183
	Uro A-sulfate isomer 2	1.86	307	*227*	183
	Uro A-disulfate	3.86	387	*307*	227

^1^ MS^2^ Ions in italic were those subjected to MS^3^ fragmentation.

## Abbreviations

The following abbreviations are used in this manuscript:

CHD	coronary heart disease
CVD	cardiovascular disease
eNOS	endothelial nitric oxide synthase
FMD	flow-mediated vasodilation
HAEC	human aortic endothelial cells
HUVEC	human umbilical vein endothelial cells
NO	nitric oxide
UHPLC-MS^n^	ultra-high performance liquid chromatography coupled to mass spectrometry
Uro A	urolithin A
Uro B	urolithin B
Uro Bgluc	urolithin B-glucuronide
